# Supplementary material for: The impact of post-hospital remote monitoring of COVID-19 patients using pulse oximetry: A national observational study using hospital activity data
Source: eClinicalMedicine. 2022 May 12;48:101441. doi: 10.1016/j.eclinm.2022.101441 (PMC9098201; doi:10.1016/j.eclinm.2022.101441)
Supplement: Supplementary file 1 [file mmc1.docx]

Supplementary figure and results of sensitivity analyses
