# Supplementary material for: The impact of post-hospital remote monitoring of COVID-19 patients using pulse oximetry: A national observational study using hospital activity data
Source: eClinicalMedicine. 2022 May 12;48:101441. doi: 10.1016/j.eclinm.2022.101441 (PMC9098201; doi:10.1016/j.eclinm.2022.101441)
Supplement: Supplementary file 2 [file mmc2.pdf]

The impact of post-hospital monitoring of COVID-19 patients using pulse oximetry: a national observational study using hospital activity data.

Supplementary material

Contents

Figure S1.	Length of stay distribution for included Covid-19 spells.	Page 2
Table S1.	Sensitivity analyses: variants of analytical models.	Page 2
Table S2.	Sensitivity analyses 2: effect of adding adjustment variables in turn	Page 3

Figure S1. Length of stay distribution for included Covid-19 spells, split by whether CVW was available or not; proportion (a) and cumulative proportions (b) of each group. Of the discharges where no CVW was available 0.79% had LOS > 60 days. For discharges with CVW available 0.92% had LOS > 60 days. All such discharges were set to 60 days in our models.

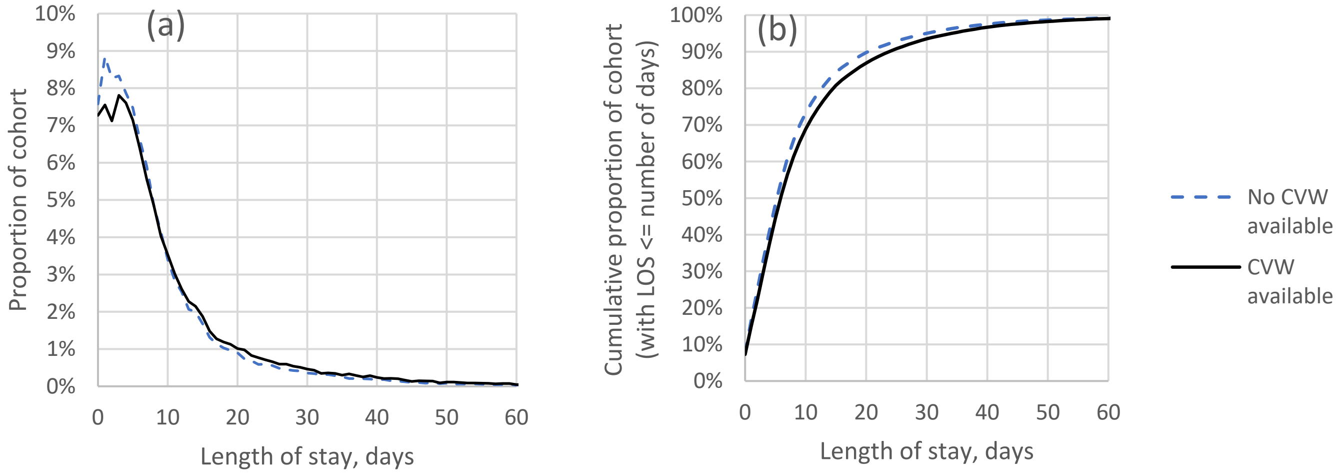

Table S1. Sensitivity analyses: variants of analytical models. Adjusted Incidence Rate Ratios (IRR) for LOS of COVID-19 spell, and adjusted Odds Ratios (OR) of COVID-19 readmissions within 28 days: discharges with CVW available, vs not available.

	**Length of stay of COVID-19 spell**				**Readmission for COVID-19 within 28 days**			
	**IRR**	**P value**	**IRR 95% confidence interval**		**OR**	**P value**	**OR 95% confidence interval**	
			**Lower**	**Upper**			**Lower**	**Upper**
Base models (figs 2 and 3)	1.05	0.01	1.01	1.09	0.97	0.32	0.91	1.03
Time period: 7 days	1.04	0.07	1.00	1.08	0.98	0.44	0.92	1.04
Time period: 28 days	1.08	0.00	1.04	1.12	0.95	0.14	0.90	1.02
Add Wave 1 data	1.01	0.62	0.97	1.05	0.99	0.76	0.92	1.07
LOS untrimmed at 60 days	1.04	0.04	1.00	1.08	-	-	-	-
LOS disregarding pre-Covid diagnosis	1.05	0.00	1.02	1.09	-	-	-	-

Table S2. Sensitivity analyses 2: Effect of adding adjustment variables in turn. Adjusted Incidence Rate Ratios (IRR) for LOS of COVID-19 spell, and adjusted Odds Ratios (OR) of COVID-19 readmissions within 28 days: discharges with CVW available, vs not available. Only one ordering shown.

	Length of stay of COVID-19 spell				Readmission for COVID-19 within 28 days			
	IRR	P value	IRR 95% confidence interval		OR	P value	OR 95% confidence interval	
			Lower	Upper			Lower	Upper
Unadjusted	1.13	<.0001	1.11	1.14	0.98	0.14	0.95	1.01
Adjusted: By time period	1.00	0.88	0.99	1.01	1.03	0.14	0.99	1.06
+ (Cluster by provider trust)	1.05	0.03	1.00	1.10	0.98	0.50	0.92	1.04
+ Age	1.05	0.02	1.01	1.09	0.99	0.67	0.93	1.05
+ Charlson	1.05	0.03	1.01	1.09	0.98	0.56	0.92	1.05
+ Deprivation +Ethnic group +Gender	1.05	0.02	1.01	1.09	0.98	0.47	0.92	1.04
+ Emergency +First +Proportion of beds occupied COVID	1.05	0.01	1.01	1.09	0.97	0.32	0.91	1.03
